# Identification of Poliovirus Receptor-like 3 Protein as a Prognostic Factor in Triple-Negative Breast Cancer

**DOI:** 10.3390/cells13151299

**Published:** 2024-08-03

**Authors:** Gian Marco Leone, Katia Mangano, Salvatore Caponnetto, Paolo Fagone, Ferdinando Nicoletti

**Affiliations:** 1 Department of Biomedical and Biotechnological Sciences, University of Catania, Via S. Sofia 97, 95123 Catania, Italy; g.marco-94@outlook.it (G.M.L.); katia.mangano@unict.it (K.M.); ferdinic@unict.it (F.N.); 2 Medical Oncology Unit B, Department of Radiology, Oncology and Pathology, Policlinico Umberto I, Sapienza University of Rome, 00161 Rome, Italy; salvo.caponnetto@uniroma1.it

**Keywords:** triple-negative breast cancer, PVRL3, EZH2, immune checkpoint, biomarker

## Abstract

Triple-negative breast cancer (TNBC) represents an aggressive subtype of breast cancer, with a bad prognosis and lack of targeted therapeutic options. Characterized by the absence of estrogen receptors, progesterone receptors, and HER2 expression, TNBC is often associated with a significantly lower survival rate compared to other breast cancer subtypes. Our study aimed to explore the prognostic significance of 83 immune-related genes, by using transcriptomic data from the TCGA database. Our analysis identified the Poliovirus Receptor-Like 3 protein (PVRL3) as a critical negative prognostic marker in TNBC patients. Furthermore, we found that the Enhancer of Zeste Homolog 2 (EZH2), a well-known epigenetic regulator, plays a pivotal role in modulating PVRL3 levels in TNBC cancer cell lines expressing EZH2 along with high levels of PVRL3. The elucidation of the EZH2-PVRL3 regulatory axis provides valuable insights into the molecular mechanisms underlying TNBC aggressiveness and opens up potential pathways for personalized therapeutic intervention.

## 1. Introduction

Breast cancer (BC) is the most commonly diagnosed malignancy and the leading cause of cancer-related death in women with approximately 2.3 million newly diagnosed cases and 700.000 death, in 2020 [1]. 

BC is categorized into four molecular subtypes, i.e., Luminal A, Luminal B, HER2-enriched, and triple-negative breast cancer (TNBC), based on the presence or absence of hormone receptors (estrogen and progesterone receptors) and the overexpression of the human epidermal growth factor receptor 2 (HER2). These markers are not just prognostic but have implications for patient management, guiding the choice of chemotherapy and targeted therapy [2]. 

Triple-negative breast cancer (TNBC) represents the most aggressive subtype of BC, characterized by the absence of estrogen receptors, progesterone receptors, and the human epidermal growth factor receptor-2 (HER2), which are usually targeted in therapy for the more common BC subtypes [3,4]. The overall survival rate at 5 years ranges from 65% to 11%, and approximately 23% of the patients develop metastases within 3 years, with a median overall survival of 12–18 months following the onset of metastasis [5,6,7]. 

In recent years, significant advancements have occurred in immunotherapy, and accumulating data from both preclinical and clinical studies suggest new potential therapeutic approaches for TNBC patients [8]. Today, there is a general consensus that TNBC generates a highly immunosuppressive microenvironment [9,10]. However, only 20% of metastatic TNBC patients positive for the Programmed Death-Ligand 1 (PD-L1) show benefits from treatment with PD-L1 inhibitors [11]. For this reason, it is of fundamental importance to find new potential immune checkpoints that can be used as therapeutic targets in TNBC. 

In light of this, our study focused on examining the expression levels of 83 immune-related genes in TNBC to uncover their prognostic significance. Our analysis identified PVRL3, also known as CD113 or Nectin3, as a gene of particular interest due to its negative prognostic implications. PVRL3, belonging to the immunoglobulin-like cellular adhesion molecule family, plays a critical role in Ca^2+^-independent cellular adhesion and is involved in the regulation of the immune system through its interaction with the TIGIT receptor on T cells and Natural Killer (NK) cells.

Recent studies have indicated that PVRL3 can influence tumor growth and metastasis by modulation of intercellular connections and cell signaling. In particular, in different cancer types (ovarian carcinoma, nasopharyngeal carcinoma, prostate carcinoma and lung adenocarcinoma), the altered expression of PVRL3 has been observed to contribute to the invasive capacity of tumor cells, facilitating their dissemination to other tissues and leading to a worse prognosis [12,13,14,15]. Additionally, evidence suggests that PVRL3 may play a role in the tumor microenvironment, influencing the local immune response and interaction with stromal cells [16]. 

Moreover, our investigation into the regulatory mechanisms of PVRL3 revealed the involvement of the Enhancer of Zeste Homolog 2 (EZH2), a component of the polycomb repressive complex 2 (PRC2) known for its role in chromatin remodeling and gene silencing, which contributes to cancer progression and metastasis.

EZH2 has been extensively studied in the context of cancer, where it is often overexpressed and associated with a worse prognosis. The overexpression of EZH2 promotes tumor metabolic profile, cell proliferation, invasiveness, and the ability to metastasize, making it a key marker of tumor progression [17,18,19]. EZH2 also contributes to treatment resistance, including chemotherapy and targeted therapies, through the epigenetic regulation of genes involved in cell survival and stress response [20,21]. Recent research has suggested that inhibiting EZH2 could represent a promising therapeutic strategy for various types of cancer, leading to the development of specific EZH2 inhibitors currently in clinical trials [22].

The association between PVRL3 and EZH2 suggests a complex network of interactions influencing tumor growth and immune evasion, offering new insights into the molecular mechanisms underlying TNBC and suggesting novel potential avenues for intervention.

## 2. Materials and Methods

### 2.1. Profiling of Immune-Related Genes in TNBC Patients

We have selected eighty-three immune-related genes mainly expressed on tumor cells and previously associated with a prognostic value in different tumors, as per publications by Li et al., 2016 [23], and Thorsson et al., 2018 [24]. The genes of interest were divided into four groups based on their role in regulating the immune response to tumors as follows: co-stimulator genes (BTN2A1, BTN2A2, BTN3A1, BTNL2, BTNL3, BTNL8, CD111, CD276, CD40LG, CD48, CD70, CD80, CD86, CXCR4, GITRL, HHLA2, LSECtin, MICA, MICB, OX40L, PVR, RAET1E, RAET1G, RAET1L, SELP, STING1, ULBP1, ULBP2, and ULBP3); embryonal antigens (CTAG2, CTCFL, DDX53, GAGE1, MAGEA1, MAGEA3, MAGEA4, MAGEA5P, MAGEA6, MAGEA8, MAGEA10, MAGEA12, MAGEB1, MAGEB10, MAGEB16, MAGEB18, MAGEB2, MAGEB4, MAGEB6, MAGEC1, MAGEC2, MAGEC3, MAGED1, MAGED2, MAGEE1, MAGEE2, MAGEF1, MAGEH1, MAGEF1, MAGEH1, MOK, and PRAME); inhibitor genes (BTN3A1, BTN3A2, CD200, CD274, CD47, IDO1, IL10, LGALS3, LGALS9, PDCD1LG2, PVRL2, PVRL3, TGFB1, and VTCN1), and MHC class I molecules (B2M, HLA-A, HLA-B, HLA-C, HLA-E, HLA-F, HLA-G, and TAPBP). To assess whether the expression levels of immune-related genes in TNBC could be used as prognostic indicators or correlated with significant clinical features, RSEM-normalized RNA Seq data from The Cancer Genome Atlas (TCGA) database were retrieved using the cBioportal web-based tool (http://www.cbioportal.org; accessed on 15 January 2024). Specifically, the Firehose Legacy TCGA dataset was selected to analyze data from 110 primary TNBC female patients without prior neoadjuvant therapy. TNBC purity index data were retrieved from the publication by Li et al., 2016 [23]. The adjustment of the gene expression data based on tumor purity and the survival analysis were performed using SPSS software (v. 28.0.1.1).

### 2.2. Cell Line and Culture

HCC1937 (CRL-2336), MDA-MB-231 (HTB-26), MDA-MB-436 (HTB-130) and MDA-MB-453 (HTB-131) human triple-negative breast cancer cell lines were purchased from the American Type Culture Collection (ATCC). All cell lines were cultured in Roswell Park Memorial Institute medium (RPMI-1640) (ATCC 30-2001), supplemented with 10% of heat-inactivated fetal bovine serum (FBS) (Corning 35-016-CV), 100 IU/mL of penicillin, 100 μg/mL of streptomycin, and 2 mM of L-glutamine (Corning, 25-005-CI), to form the complete culture medium formulation. The cells were maintained at 37 °C in a humified chamber with 5% CO_2_ and 95% relative humidity.

### 2.3. Drug Treatments

The drugs used in this study were as follows: BIX01294 (B9311, Sigma-Aldrich, Darmstadt, Germany), GSK343 (SML0766, Sigma-Aldrich), GSK2801 (SML0768, Sigma-Aldrich), JQ1 (SML0974, Sigma-Aldrich), and Vorinostat (SML0061, Sigma-Aldrich).

### 2.4. Cell Viability

The 3-(4,5-Dimethylthiazol-2-yl)-2,5-diphenyl tetrazolium bromide or MTT (Sigma-Aldrich) colorimetric viability assay was used to measure the metabolic activity as an indicator of cellular viability. 

For treatments, MDA-MB-231 cells (5 × 10^4^/well) were treated with 8 increasing doses of each drug (0.01 nM to 100 uM) for 72 h. The viability of the cells was evaluated by introducing 0.5 µg/mL of MTT into each well and the plates were further incubated at 37 °C for 3 h. Thereafter, the medium was removed and 100 μL of DMSO was added to each well to dissolve insoluble purple formazan crystals. The resulting optical density (OD) was quantified at 570 nm using spectrophotometric analysis (Tecan Sunrise Microplate Reader). The untreated (mock-treated) controls were considered 100% viable. 

For the dose–response curves, the obtained percentage viability values were plotted using Graph-Pad Prism (v.7) software, and the relative IC50 values were calculated.

### 2.5. Generation of Human TNBC Cell Lines Constitutively Overexpressing EZH2

To generate human TNBC cell lines that overexpress EZH2, human EZH2 was amplified utilizing cDNA sourced from MDA-MB-231 cells and subsequently inserted into the pcDNA3.2/GW/D-TOPO expression vector (Thermo Fisher, Waltham, MA, USA). The corresponding empty expression vector was used as a control. Transfection of the expression vectors into MDA-MB-231 cells was performed using Lipofectamine 2000 reagent (Invitrogen, Waltham, MA, USA) following the manufacturer’s guidelines. In detail, 3 × 10^6^ cells were seeded in a 6-well plate. The day after, the cells were transfected using Lipofectamine 2000 transfection reagent and then incubated overnight at 37 °C, in a humified chamber. The day after, neomycin (N1142, Sigma-Aldrich) was added to the cells and diluted in fresh complete culture medium, at 1 µg/mL final. The cells were then maintained in 1 µg/mL neomycin selective complete medium for one week before the validation of EZH2 overexpression using RT-qPCR analysis.

### 2.6. Generation of Human TNBC Cell Lines Silenced for EZH2

To generate human TNBC cell lines constitutively silenced for EZH2, a small interfering RNA (siRNA) strategy was used. The EZH2-silencing siRNA vector was prepared via transfecting 3 × 10^6^ of each cell line with 100 nM of validated siRNA for EZH2 (6509, Cell Signaling Technology, Danvers, MA, USA) or a negative control (6568, Cell Signaling Technology), using Lipofectamine 2000 reagent following the manufacturer’s instructions. After 72 h of transfection, the medium was replaced with a fresh complete medium. The successful silencing of EZH2 was confirmed using RT-qPCR analysis by assessing the expression of EZH2.

### 2.7. Total RNA Extraction, cDNA Synthesis, and Quantitative RT-qPCR Analyses

Total RNA extraction was performed using GeneJET RNA Purification Kit (K0702, Thermo Fisher Scientific), according to the manufacturer’s protocol for mammalian cultured suspension cells. 

The quantification and quality control of the obtained RNA was performed by using the NanoDrop Microvolume Spectrophotometer (Thermo Fisher Scientific). For reverse transcription, 3 μg of total RNA was retrotranscribed into cDNA, using Super-Script IV Reverse Transcriptase kit (18090050, Thermo Fisher Scientific), following the manufacturer’s instructions. The primers specific for Human EZH2, PVRL3, and GAPDH (Table 1) were designed using the Primer-Blast priming design tool provided by NCBI. 

For quantitative RT-qPCR, the Luminaris Color HiGreen qPCR Master Mix high ROX 2X (K0362, Thermo Fisher Scientific) was used under the following cycling condition: UDG pre-treatment at 50 °C for 2 min and an initial denaturation at 95 °C for 10 min; and 40 cycles which comprise denaturation at 95 °C for 15 s, annealing at 60 °C for 30 s, and extension at 72 °C for 30 s. EZH2 and PVRL3 gene expression levels were assessed and normalized to the Human GAPDH constitutive gene. The relative expression was determined using the formula 2^−∆∆Ct,^ and the findings are presented as the mean gene expression ± standard deviation (SD).

### 2.8. Computational Analysis for Regulation of PVRL3 Expression

To explore biological mechanisms involved in the regulation of the PVRL3 gene, we identified the transcriptional factors that can interact with the promoter regions of PVRL3 by interrogating the Enrichr database [25]. Additionally, we utilized the ARCHS4 RNA-Seq database to identify the top 100 genes that exhibited the highest correlation with PVRL3. By identifying the top 100 genes exhibiting the strongest correlation with PVRL3, we aimed to uncover potential regulatory networks that could shed light on the intricate molecular mechanisms governing PVRL3 expression. 

Following the identification of these highly correlated genes, the EnrichR database (accessed on 18 January 2024) was used to predict the putative transcription factors responsible for orchestrating their expression. The transcriptional factors with the highest combined score and adjusted *p*-value < 0.05 were considered as genes involved in the regulation of the PVRL3 gene. 

### 2.9. Statistical Analyses

The TCGA gene expression data were analyzed using a General Linear Model to adjust for tumor purity levels. For survival analysis, the samples were categorized based on the expression levels of each gene of interest. Specifically, samples in the upper and lower quartiles were chosen as comparison groups. Survival analysis was performed using the Cox Proportional Hazards Regression Model, with the stage of the disease included as a covariate. 

The experiments on TNBC cell lines were conducted as three independent sets, each comprising three technical replicates. The results are presented as mean ± standard deviation (S.D.). Shapiro–Wilk and Kolmogorov–Smirnov tests were used to assess the normality distribution of the data. Comparisons between groups were conducted using the ANOVA test followed by the Tukey’s test. A *p* value < 0.05 was considered as threshold for statistical significance. PRISM and SPSS statistical analysis software were employed for data analysis and figure generation. 

## 3. Results

### 3.1. Prognostic Relevance of Immune-Related Genes in TNBC

**Figure 1:**
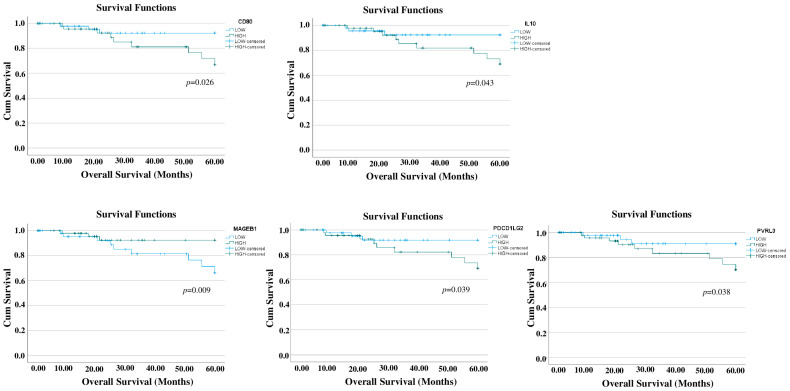
Survival analysis. The TCGA TNBC patients were stratified in quartiles based on the expression of the 83 genes of interest, following normalization for tumor purity, and samples in the upper and lower quartiles were selected for comparison. Cox Proportional Hazards Regression Model, with the stage of the disease included as a covariate, was used to assess the prognostic values of each gene. Kaplan–Meier curves only for the statistically significant genes are shown.

To explore the clinical relevance of the selected 83 selected genes, Kaplan–Meier analyses were performed for all selected genes. The TCGA TNBC patients were stratified in quartiles based on the expression of the genes of interest, following normalization for tumor purity, and samples in the upper and lower quartiles were selected for the comparison. As shown in Figure 1, higher expression levels of MAGEB1 are associated with a significantly higher overall survival (*p* = 0.009). On the other hand, higher expression levels of IL10 (*p* value = 0.043), CD80 (*p* = 0.026), PDCD1LG2 (*p* = 0.038), and PVRL3 (*p* = 0.039) are associated with a significantly lower overall survival. No significance was observed for the other selected genes. 

### 3.2. PVRL3 Gene Expression Is Higher in TNBC Cell Lines

**Figure 2:**
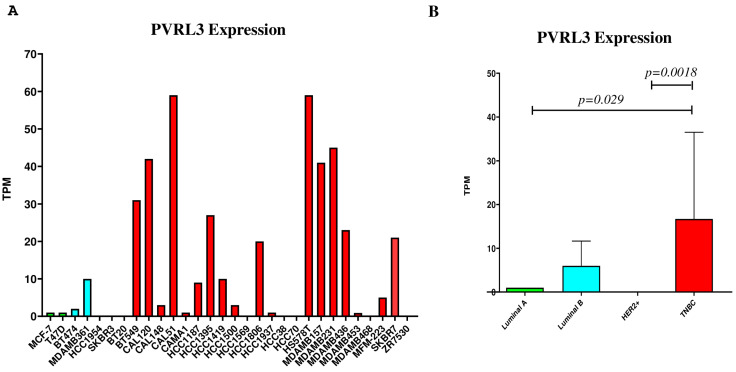
PVRL3 expression levels in 28 breast cancer cell lines (**A**); PVRL3 expression in breast cancer cell lines aggregated based on the PAM50 molecular classification of breast tumors (https://www.ebi.ac.uk/gxa/experiments/E-MTAB-4801/Results, accessed on 1 April 2024) (**B**). Comparisons between two groups on a single parameter were conducted using the two-tailed unpaired *t*-test.

To investigate the expression of PVRL3 in BC, a comprehensive expression analysis was conducted across 30 BC cell lines. These included two Luminal A, two Luminal B, two HER2-positive, and twenty-four TNBC lines, utilizing data sourced from the Expression Atlas database (https://www.ebi.ac.uk/gxa/home, accessed on 8 January 2024) (Figure 2A). Our analysis revealed that PVRL3 expression levels were higher in TNBC cell lines compared to those in the other BC categories under investigation. This difference in expression levels reached the statistical significance when comparing TNBC to Luminal A (*p* = 0.029) and HER2-enriched (*p* = 0.0018) tumors (Figure 2B). 

### 3.3. The Inhibition of EZH2 Catalytic Activity Modulates PVRL3 Expression in MDA-MB-231 Cells

**Figure 3:**
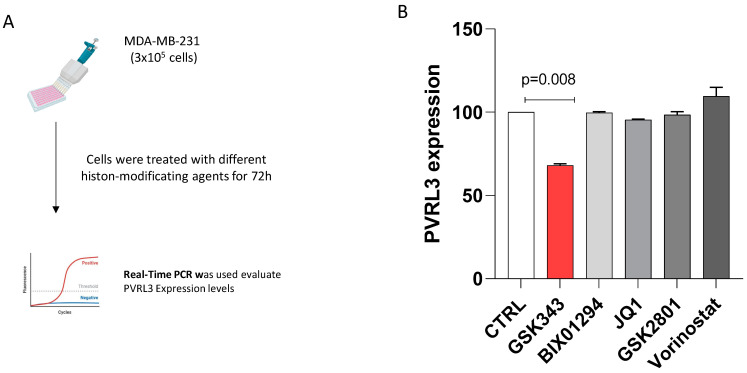
Study workflow (**A**); PVRL3 expression levels in MDA-MB-231 cell line after 72 h of treatment with DMSO (CTRL), BIX01294, GSK343, GSK2801, JQ1, and Vorinostat (**B**).

To investigate the role of epigenetic regulation on the expression of the PVRL3 gene, MDA-MB-231 cells were treated with a range of compounds known to affect histone modifications (Figure 3A). These drugs included BIX01294 (an inhibitor of the euchromatic histone-lysine N-methyltransferase 2 (EHMT2) that acts on the methylation of lysine 9 of histone H3), GSK343 (an inhibitor of EZH2 that acts on the methylation of lysine 27 of the histone H3), JQ1 (an inhibitor of bromodomain-containing protein 4—BRD4—that acts on the methylation of lysine 4 of histone H3 and on the acetylation of lysine 27 of the histone H3), GSK2801 (an inhibitor of bromodomain adjacent to zinc finger domain, BAZ2a and BAZ2b, that acts on the acetylation of lysine 14 and lysine 27 of histone H3), and Vorinostat (an inhibitor of class I, II, and IV of histone deacetylases). The IC50 values obtained in the MDA-MB-231 cell line were 3 μM for BIX01294, 2 μM for GSK343, 100 nM for JQ1, 10 μM for GSK2801, and 5 μM for Vorinostat. Subsequently, to assess the drug effect on the expression of the PVRL3 gene, all cells were treated with the IC50 values for each drug, respectively. Total RNA was extracted from the samples, and the expression level of the PVRL3 gene was assessed by RT-qPCR. As shown in Figure 3B, PVRL3 expression level significantly decreased (about 30%) after the treatment with 2 μM GSK343, which acts as an inhibitor of EZH2 (Figure 3B). On the other hand, no significant alteration in PVRL3 expression was observed after the treatment with other inhibitors of histone modifications (Figure 3B). 

### 3.4. PVRL3 Expression Is Modulated by EZH2

**Figure 4:**
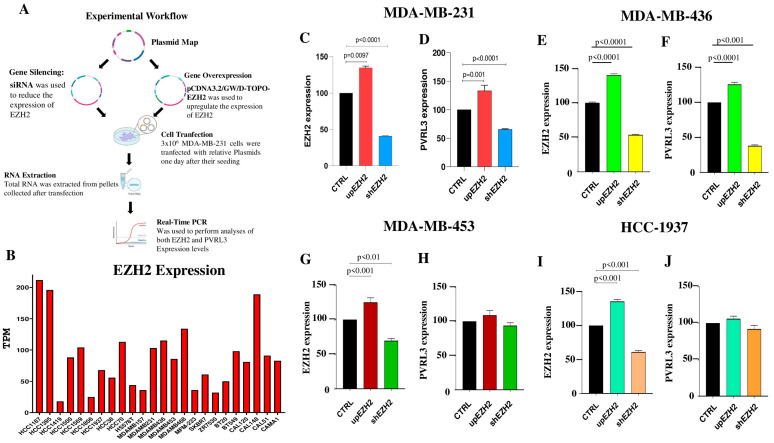
Study workflow (**A**). EZH2 expression levels in 24 TNBC cell lines (**B**). Modulation of EZH2 and PVRL3 expression in MDA-MB-231 in control cells (CTRL) upon overexpression (upEZH2) and following EZH2 silencing (shEZH2) (**C**,**D**). Modulation of EZH2 and PVRL3 expression in MDA-MB-436 in control cells (CTRL) upon overexpression (upEZH2) and following EZH2 silencing (shEZH2) (**E**,**F**). Modulation of PVRL3 expression in MDA-MB-453 in control cells (CTRL) upon overexpression (upEZH2) and following EZH2 silencing (shEZH2) (**G**,**H**). Modulation of PVRL3 expression in HCC1937 in control cells (CTRL) upon overexpression (upEZH2) and following EZH2 silencing (shEZH2) (**I**,**J**).

Based on the results obtained with GSK343, we aimed to study the effect of EZH2 modulation on PVRL3 expression in different TNBC cell lines. As shown in Figure 4A, we employed two complementary approaches (Figure 4A). In the first approach, TNBC cell lines were transfected with pCDNA3.2/GW/D-TOPO-EZH2 plasmid in order to induce the expression of EZH2. In the second approach, the cell lines were transfected with 100 nM of siRNA for EZH2 to assess whether the downregulation of EZH2 expression was associated with a reduction in PVRL3 mRNA levels (Figure 4A). For this analysis, we selected two cell lines showing high levels of PVRL3 (MDA-MB-231 and MDA-MB-436), and two cell lines expressing low levels of PVRL3 (HCC1937 and MDA-MB-453). As shown in Figure 4B, the four selected TNBC cell lines expressed EZH2 (Figure 4B).

The expression of EZH2 was evaluated after plasmid and siRNA transfections by using RT-qPCR. As shown in Figure 4C,E,G,I, the genetic manipulation of HCC1937, MDA-MB-231, MDA-MB-436, and MDA-MB-453 cells was successful, with a 35%, 35%, 40%, and 25% increase in EZH2 transcript expression in upEZH2 versus relative CTRL (*p* = 0.0097), respectively, and a 54%, 60%, 42%, and 30% reduction in mRNA expression in shEZH2 compared to the expression in CTRL, respectively (Figure 4C,E,G,I). RT-qPCR analyses for PVRL3 revealed a statistically significant upregulation of PVRL3 in MDA-MB-231 and MDA-MB-436 cells upon EZH2 overexpression (*p* = 0.001 and *p* < 0.001, respectively). Accordingly, silencing of EZH2 was associated with a significant reduction in PVRL3 transcript levels in MDA-MB-231 and MDA-MB-453 cells (*p* < 0.0001 for both cell lines) (Figure 4D,F). On the other hand, no statistically significant modulation of PVRL3 levels was observed in HCC1937 and MDA-MB-453 cells (Figure 4H,J). 

### 3.5. Computational Analysis Identifies EZH2 as a Regulator Factor of PVRL3

**Figure 5:**
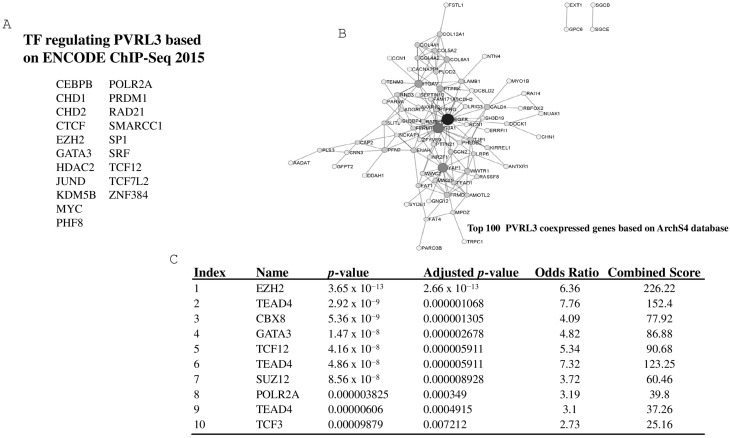
The 20 potential TFs (transcription factors) capable of binding to different PVRL3 promoter regions (**A**). Top 100 PVRL3 co-expressed genes based on Arch4 database (**B**). Top 10 TFs regulating the top 100 PVRL3 co-expressed genes (**C**).

To confirm EZH2 as a potential factor involved in the regulation of PVRL3 gene expression, a computational analysis was conducted using the bioinformatics tool Enrichr. This analysis enabled the identification of 20 potential transcription factors capable of binding to the promoter of the PVRL3 gene (Figure 5A). Subsequently, the top 100 genes co-expressed with PVRL3 were identified using the ARCHS4 RNA-seq database (Figure 5B). To identify the main transcription factors involved in the regulation of these genes, we interrogated the ENCODE TF ChIP-seq 2015 database, implemented in Enrichr. This analysis allowed us to identify EZH2 as the most significantly enriched transcription factor (adjusted *p*-value = 2.665 × 10^−13^) (Figure 5C). 

## 4. Discussion

TNBC is a highly aggressive tumor type characterized by limited therapeutic options. The immune system and the interaction with the tumor microenvironment play a fundamental role in tumor progression and therapy response. Immunotherapy has emerged as a promising approach for various tumors, especially those with an inflamed or ‘hot’ microenvironment [26,27,28]. However, the effectiveness of immunotherapy in the case of BC, particularly the triple-negative subtype, has been modest compared to other tumor types. 

In this study, we explored the role of 83 immune checkpoints expressed on the surface of tumor cells. This analysis revealed CD80, IL10, PDCD1LG2, and PVRL3 as immune-related genes with negative prognostic values. 

Conversely, MAGEB1 is identified as an immune-related gene that possesses a positive prognostic value for TNBC patients. The MAGE family, particularly the B subset to which MAGEB1 belongs, has been studied for its expression in various malignancies and its potential as a target for cancer immunotherapy [29,30]. MAGE genes typically encode for antigens that are recognized by the immune system, making them candidates for cancer vaccine development [31]. The positive prognostic value of MAGEB1 could be attributed to its ability to elicit a strong immune response, thereby aiding the body in identifying and combating tumor cells more effectively. Furthermore, the expression of MAGEB1 in tumors may serve as a biomarker for immune activity within the tumor microenvironment. A high level of MAGEB1 expression could indicate an active immune response against the tumor, contributing to a more favorable prognosis. 

There is currently a lack of scientific literature regarding the role of PVRL3 in TNBC. The limited availability of studies addressing this specific aspect of BC biology underscores the need for dedicated research efforts to elucidate the potential contributions of PVRL3 in the context of TNBC. Given the recognized heterogeneity within BC subtypes, an exploration of the specific involvement of PVRL3 in TNBC may uncover novel insights into the molecular mechanisms driving this aggressive subtype. 

PVRL3 belongs to the family of immunoglobulin-like cellular adhesion molecules, playing a significant role in calcium-independent cellular adhesion [32]. Recently, PVRL3 has been identified as a ligand for TIGIT, a known inhibitor of T and NK cell function [33]. This interaction between PVRL3 and TIGIT emphasizes its influence in modulating immune responses, hinting at possible opportunities for new treatments. The TIGIT receptor, a T-cell receptor that curtails T-cell activity and adaptive immune responses, is crucial in the evasion mechanisms of cancer. Predominantly expressed by lymphocytes, TIGIT shows varied levels of expression across different T-cell subsets, including CD4+ T cells, CD8+ T cells, regulatory T cells (Tregs), and follicular T helper cells, as well as NK cells, with the highest levels observed in Tregs, memory, and activated T cells and NK cells in healthy subjects. Structurally, TIGIT comprises an external immunoglobulin variable (IgV) domain, a type 1 transmembrane domain, and a cytoplasmic tail housing an ITIM- and an ITT-like motif, whose phosphorylation activates a signal inhibiting TIGIT upon its ligand binding. 

Studies involving TIGIT-deficient models have highlighted its vital role in cancer progression, with reduced tumor growth in TIGIT-deficient mice compared to normal counterparts, especially in breast and colon cancers, along with a notable increase in survival rates in myeloma models and protection against experimental lung metastasis. The widespread expression of the TIGIT of tumor-infiltrating lymphocytes in diseases like melanoma, pancreatic, breast, and lung cancers, among others, earmarks it as a crucial target for immune checkpoint blockade. 

The pursuit of TIGIT as a therapeutic target has led to the development of monoclonal antibodies that bind to the TIGIT receptor on T cells and NK cells, showing promising results in reducing tumor growth and enhancing survival in various cancer models, including myeloma, and providing protection against tumor metastasis and recurrence [33]. 

Pharmacological interventions targeting histone modifications revealed that GSK343, an EZH2 inhibitor, led to a notable decrease in PVRL3 expression. Functional studies involving EZH2 modulation through overexpression, siRNA-mediated silencing, and pharmacological inhibition in MDA-MB-231 and MDA-MB-436 cell lines further underscored the impact of EZH2 on PVRL3 expression levels. This comprehensive approach provides valuable insights into the regulatory network involving immune checkpoints and histone modifications, offering potential avenues for therapeutic interventions in BC. 

EZH2 is a methyltransferase and a component of the polycomb repressive complex 2 (PRC2) [34], and it is involved in catalyzing the trimethylation of histone H3 at Lys 27 (H3K27me3) [35]. This modification is pivotal in regulating gene expression by inducing chromatin compaction and transcriptional repression. However, an increasing number of studies have shown several “non-canonical” functions of EZH2 [36]. EZH2 non-canonical roles seem to depend on the context, and include activities such as methylating non-histone substrates and engaging in interactions with transcription factors outside of the PRC2 complex to enhance gene expression. For instance, EZH2 has been observed to positively regulate androgen receptor gene expression in castration-resistant prostate cancer [37]. Additionally, it has been shown that EZH2 can enhance the STAT3 signaling pathway in glioblastoma and oral squamous cell carcinoma [38,39]. 

In the context of TNBC, EZH2 plays a pivotal role in its progression by promoting the migration and invasion of cancer cells through the regulation of the TIMP2-MMP-2/-9 pathway. Specifically, EZH2 modulates the balance between tissue inhibitors of metalloproteinases (TIMP2) and matrix metalloproteinases (MMP-2 and MMP-9), which are critical for extracellular matrix remodeling. By downregulating TIMP2 and upregulating MMP-2 and MMP-9, EZH2 enhances the ability of TNBC cells to degrade the extracellular matrix, facilitating their invasive and migratory capabilities [40]. Additionally, EZH2-mediated methylation of H3K27 at the FOSB promoter suppresses its expression, which is vital in TNBC progression. When FOSB expression is suppressed by this epigenetic alteration, TNBC cells demonstrate accelerated tumor growth both in vitro and in vivo [41]. More recently, Dardis and colleagues showed that EZH2 can promote RELB and NFKB2 gene expression in TNBC. This interaction promotes the expression of various oncogenes and inflammatory mediators, which contribute to the aggressive behavior of TNBC. The EZH2-NF-κB axis not only enhances the proliferation and survival of cancer cells but also increases their invasive potential, making it a significant driver of tumor growth and dissemination [18]. 

The multiple involvement of EZH2 is supported by a growing body of evidence from both in vitro and in vivo models, as well as clinical studies. Notably, EZH2 has been implicated in various biological mechanisms, including the regulation of the cell cycle, facilitation of cell migration, modulation of apoptosis, and contribution to drug resistance [42,43,44,45]. This multifunctional role positions EZH2 as a significant player in diverse cellular processes, with implications for both normal physiology and pathological conditions, such as cancer.

Based on our data, the efficacy of combining EZH2 inhibitors with inhibitors targeting the PVRL3-TIGIT axis appears to be contingent upon the expression levels of PVRL3 and EZH2. Specifically, only patients whose tumors express both PVRL3 and EZH2 are likely to benefit from this therapeutic strategy. Indeed, we have observed that only cell lines expressing EZH2 along with high levels of PVLR3 respond to EZH2 modulation by downregulating PVRL3.

This point underscores the importance of biomarker expression levels in determining the suitability and potential success of targeted cancer therapies. By identifying patients with the appropriate molecular profiles, clinicians can personalize treatment plans to improve outcomes and avoid ineffective interventions.

Our study has some limitations that must be acknowledged. Firstly, all our analyses are limited to mRNA data, and no protein-level validation has been performed. Therefore, while we can infer changes in gene expression, we cannot confirm whether these changes translate to corresponding alterations in protein abundance or activity, which is crucial for understanding the functional impact of gene expression changes. Secondly, we did not correct for multiple comparisons in our survival analyses. Given the limited cohort size, implementing such corrections could significantly reduce statistical power, making it more challenging to detect significant effects. Additionally, our attempts to replicate our findings using a comparable TNBC cohort from the Gene Expression Omnibus (GEO) dataset were unsuccessful, as no suitable dataset with overlapping clinical characteristics and survival data was available. This limitation restricts the generalizability of our results. All of these constraints underscore the need for future research to include proteomic validation and explore larger, more comprehensive datasets to enhance the robustness and applicability of our findings.

## 5. Conclusions

In summary, our study enhances the understanding of the immune landscape within TNBC and its implications for immunotherapy. Different immune checkpoints can be expressed on the surface of tumor cells enhancing the immune evasion of tumor. We identified PVRL3 as an immune checkpoint with a negative prognostic role in TNBC. Identifying EZH2 as a regulator of PVRL3 also makes it a potential target to enhance immunotherapy, reducing immune evasion. This is particularly important in light of the fact that several small-molecule inhibitors targeting EZH2 have been developed and tested in clinical trials. Notably, Tazemetostat (EPZ-6438, marketed as Tazverik) is currently approved by the FDA for treating epithelioid sarcoma and follicular lymphoma. Also, Constellation Pharmaceuticals has developed two EZH2 inhibitors, CPI-1205 and CPI-0209, which are being evaluated in phase 1/2 clinical trials in combination with other drugs for treating metastatic castration-resistant prostate cancer (mCRPC). Other EZH2 inhibitors undergoing clinical trials include SHR2554 and PF-06821497. Additionally, Daiichi Sankyo’s Valemotostat (DS-3201b), an EZH1/2 inhibitor, is undergoing phase 1/2 clinical trials for various hematological and solid tumors [46]. 

Further investigations are needed to clarify the underlying mechanisms that affect the immune landscape in this tumor subtype and to develop tailored immunotherapeutic approaches that harness the potential of the immune system to effectively fight TNBC, paving the way for more effective immunotherapies in this tumor.

## Figures and Tables

**Table 1:** Primer sequences used in RT-qPCR.

Target Transcript	Primer Name	Sequence (5′->3′)	Product Length (bp)
PVRL3	PVRL3_F	GCAGTTCACCATCCCCAATATG	162
	PVRL3_R	TCCAAGCGGGAATGTAACAGC	
EZH2	EZH2_F	AATCAGAGTACATGCGACTGAGA	141
	EZH2_R	GCTGTATCCTTCGCTGTTTCC	
GAPDH	GAPDH_F	AGAAGGCTGGGGCTCATTTG	258
	GAPDH_R	AGGGGCCATCCACAGTCTTC	

## Data Availability

The authors confirm that the data supporting the findings of this study are available within the article. Further data that support the findings of this study are available on the Expression Atlas database (https://www.ebi.ac.uk/gxa/home), Enrichr database (http://maayanlab.cloud/Enrichr/enrich) and cBioportal web-based tool (http://www.cbioportal.org).

## References

[1] Sung H., Ferlay J., Siegel R.L., Laversanne M., Soerjomataram I., Jemal A., Bray F. (2021). Global Cancer Statistics 2020: GLOBOCAN Estimates of Incidence and Mortality Worldwide for 36 Cancers in 185 Countries. CA Cancer J. Clin..

[2] Feng Y., Spezia M., Huang S., Yuan C., Zeng Z., Zhang L., Ji X., Liu W., Huang B., Luo W. (2018). Breast Cancer Development and Progression: Risk Factors, Cancer Stem Cells, Signaling Pathways, Genomics, and Molecular Pathogenesis. Genes Dis..

[3] Derakhshan F., Reis-Filho J.S. (2022). Pathogenesis of Triple-Negative Breast Cancer. Annu. Rev. Pathol. Mech. Dis..

[4] Ensenyat-Mendez M., Llinàs-Arias P., Orozco J.I.J., Íñiguez-Muñoz S., Salomon M.P., Sesé B., DiNome M.L., Marzese D.M. (2021). Current Triple-Negative Breast Cancer Subtypes: Dissecting the Most Aggressive Form of Breast Cancer. Front. Oncol..

[5] Won K., Spruck C. (2020). Triple-negative Breast Cancer Therapy: Current and Future Perspectives (Review). Int. J. Oncol..

[6] Baranova A., Krasnoselskyi M., Starikov V., Kartashov S., Zhulkevych I., Vlasenko V., Oleshko K., Bilodid O., Sadchikova M., Vinnyk Y. (2022). Triple-Negative Breast Cancer: Current Treatment Strategies and Factors of Negative Prognosis. J. Med. Life.

[7] Huppert L.A., Gumusay O., Rugo H.S. (2022). Emerging Treatment Strategies for Metastatic Triple-Negative Breast Cancer. Ther. Adv. Med. Oncol..

[8] Li L., Zhang F., Liu Z., Fan Z. (2023). Immunotherapy for Triple-Negative Breast Cancer: Combination Strategies to Improve Outcome. Cancers.

[9] Keren L., Bosse M., Marquez D., Angoshtari R., Jain S., Varma S., Yang S.-R., Kurian A., Van Valen D., West R. (2018). A Structured Tumor-Immune Microenvironment in Triple Negative Breast Cancer Revealed by Multiplexed Ion Beam Imaging. Cell.

[10] Zheng Y., Li S., Tang H., Meng X., Zheng Q. (2023). Molecular Mechanisms of Immunotherapy Resistance in Triple-Negative Breast Cancer. Front. Immunol..

[11] Kwa M.J., Adams S. (2018). Checkpoint Inhibitors in Triple-negative Breast Cancer (TNBC): Where to Go from Here. Cancer.

[12] Maniwa Y., Nishio W., Okita Y., Yoshimura M. (2012). Expression of Nectin 3: Novel Prognostic Marker of Lung Adenocarcinoma. Thorac. Cancer.

[13] Zhao Y., Hong X., Li K., Li Y., Li Y., He S., Zhang P., Li J., Li Q., Liang Y. (2020). ZNF582 Hypermethylation Promotes Metastasis of Nasopharyngeal Carcinoma by Regulating the Transcription of Adhesion Molecules Nectin-3 and NRXN3. Cancer Commun..

[14] Xu F., Si X., Wang J., Yang A., Qin T., Yang Y. (2019). Nectin-3 Is a New Biomarker That Mediates the Upregulation of MMP2 and MMP9 in Ovarian Cancer Cells. Biomed. Pharmacother..

[15] Suhovskih A.V., Kashuba V.I., Klein G., Grigorieva E.V. (2017). Prostate Cancer Cells Specifically Reorganize Epithelial Cell-Fibroblast Communication through Proteoglycan and Junction Pathways. Cell Adh. Migr..

[16] Wu B., Zhong C., Lang Q., Liang Z., Zhang Y., Zhao X., Yu Y., Zhang H., Xu F., Tian Y. (2021). Poliovirus Receptor (PVR)-like Protein Cosignaling Network: New Opportunities for Cancer Immunotherapy. J. Exp. Clin. Cancer Res..

[17] Zhang T., Gong Y., Meng H., Li C., Xue L. (2020). Symphony of Epigenetic and Metabolic Regulation—Interaction between the Histone Methyltransferase EZH2 and Metabolism of Tumor. Clin. Epigenet..

[18] Dardis G.J., Wang J., Simon J.M., Wang G.G., Baldwin A.S. (2023). An EZH2-NF-ΚB Regulatory Axis Drives Expression of pro-Oncogenic Gene Signatures in Triple Negative Breast Cancer. iScience.

[19] Sun S., Yu F., Xu D., Zheng H., Li M. (2022). EZH2, a Prominent Orchestrator of Genetic and Epigenetic Regulation of Solid Tumor Microenvironment and Immunotherapy. Biochim. Biophys. Acta-Rev. Cancer.

[20] Chen Y., Zhu H., Luo Y., Tong S., Liu Y. (2024). EZH2: The Roles in Targeted Therapy and Mechanisms of Resistance in Breast Cancer. Biomed. Pharmacother..

[21] Yu W., Liu N., Song X., Chen L., Wang M., Xiao G., Li T., Wang Z., Zhang Y. (2023). EZH2: An Accomplice of Gastric Cancer. Cancers.

[22] Tao L., Zhou Y., Luo Y., Qiu J., Xiao Y., Zou J., Zhang Y., Liu X., Yang X., Gou K. (2024). Epigenetic Regulation in Cancer Therapy: From Mechanisms to Clinical Advances. MedComm-Oncology.

[23] Li B., Severson E., Pignon J.-C., Zhao H., Li T., Novak J., Jiang P., Shen H., Aster J.C., Rodig S. (2016). Comprehensive Analyses of Tumor Immunity: Implications for Cancer Immunotherapy. Genome Biol..

[24] Thorsson V., Gibbs D.L., Brown S.D., Wolf D., Bortone D.S., Ou Yang T.-H., Porta-Pardo E., Gao G.F., Plaisier C.L., Eddy J.A. (2018). The Immune Landscape of Cancer. Immunity.

[25] Chen E.Y., Tan C.M., Kou Y., Duan Q., Wang Z., Meirelles G.V., Clark N.R., Ma’ayan A. (2013). Enrichr: Interactive and Collaborative HTML5 Gene List Enrichment Analysis Tool. BMC Bioinform..

[26] Lombardo S., Bramanti A., Ciurleo R., Basile M., Pennisi M., Bella R., Mangano K., Bramanti P., Nicoletti F., Fagone P. (2020). Profiling of Inhibitory Immune Checkpoints in Glioblastoma: Potential Pathogenetic Players. Oncol. Lett..

[27] Knight A., Karapetyan L., Kirkwood J.M. (2023). Immunotherapy in Melanoma: Recent Advances and Future Directions. Cancers.

[28] Huuhtanen J., Kasanen H., Peltola K., Lönnberg T., Glumoff V., Brück O., Dufva O., Peltonen K., Vikkula J., Jokinen E. (2023). Single-Cell Characterization of Anti–LAG-3 and Anti–PD-1 Combination Treatment in Patients with Melanoma. J. Clin. Investig..

[29] Verma S., Swain D., Kushwaha P.P., Brahmbhatt S., Gupta K., Sundi D., Gupta S. (2024). Melanoma Antigen Family A (MAGE A) as Promising Biomarkers and Therapeutic Targets in Bladder Cancer. Cancers.

[30] Li S., Shi X., Li J., Zhou X. (2021). Pathogenicity of the MAGE Family (Review). Oncol. Lett..

[31] Ai H., Yang H., Li L., Ma J., Liu K., Li Z. (2023). Cancer/Testis Antigens: Promising Immunotherapy Targets for Digestive Tract Cancers. Front. Immunol..

[32] Takai Y., Ikeda W., Ogita H., Rikitake Y. (2008). The Immunoglobulin-Like Cell Adhesion Molecule Nectin and Its Associated Protein Afadin. Annu. Rev. Cell Dev. Biol..

[33] Yu X., Harden K., C Gonzalez L., Francesco M., Chiang E., Irving B., Tom I., Ivelja S., Refino C.J., Clark H. (2009). The Surface Protein TIGIT Suppresses T Cell Activation by Promoting the Generation of Mature Immunoregulatory Dendritic Cells. Nat. Immunol..

[34] Greer E.L., Shi Y. (2012). Histone Methylation: A Dynamic Mark in Health, Disease and Inheritance. Nat. Rev. Genet..

[35] Yamagishi M., Uchimaru K. (2017). Targeting EZH2 in Cancer Therapy. Curr. Opin. Oncol..

[36] Wang J., Wang G.G. (2020). No Easy Way Out for EZH2: Its Pleiotropic, Noncanonical Effects on Gene Regulation and Cellular Function. Int. J. Mol. Sci..

[37] Davies A., Nouruzi S., Ganguli D., Namekawa T., Thaper D., Linder S., Karaoğlanoğlu F., Omur M.E., Kim S., Kobelev M. (2021). An Androgen Receptor Switch Underlies Lineage Infidelity in Treatment-Resistant Prostate Cancer. Nat. Cell Biol..

[38] Kim E., Kim M., Woo D.-H., Shin Y., Shin J., Chang N., Oh Y.T., Kim H., Rheey J., Nakano I. (2013). Phosphorylation of EZH2 Activates STAT3 Signaling via STAT3 Methylation and Promotes Tumorigenicity of Glioblastoma Stem-like Cells. Cancer Cell.

[39] Zheng M., Cao M., Luo X., Li L., Wang K., Wang S., Wang H., Tang Y., Tang Y., Liang X. (2019). EZH2 Promotes Invasion and Tumour Glycolysis by Regulating STAT3 and FoxO1 Signalling in Human OSCC Cells. J. Cell. Mol. Med..

[40] Chien Y.-C., Liu L.-C., Ye H.-Y., Wu J.-Y., Yu Y.-L. (2018). EZH2 Promotes Migration and Invasion of Triple-Negative Breast Cancer Cells via Regulating TIMP2-MMP-2/-9 Pathway. Am. J. Cancer Res..

[41] Zhang R., Li X., Liu Z., Wang Y., Zhang H., Xu H. (2020). EZH2 Inhibitors-Mediated Epigenetic Reactivation of FOSB Inhibits Triple-Negative Breast Cancer Progress. Cancer Cell Int..

[42] Nakagawa S., Okabe H., Sakamoto Y., Hayashi H., Hashimoto D., Yokoyama N., Sakamoto K., Kuroki H., Mima K., Nitta H. (2013). Enhancer of Zeste Homolog 2 (EZH2) Promotes Progression of Cholangiocarcinoma Cells by Regulating Cell Cycle and Apoptosis. Ann. Surg. Oncol..

[43] Ma D.-N., Chai Z.-T., Zhu X.-D., Zhang N., Zhan D.-H., Ye B.-G., Wang C.-H., Qin C.-D., Zhao Y.-M., Zhu W.-P. (2016). MicroRNA-26a Suppresses Epithelial-Mesenchymal Transition in Human Hepatocellular Carcinoma by Repressing Enhancer of Zeste Homolog 2. J. Hematol. Oncol..

[44] Dou D., Ge X., Wang X., Xu X., Zhang Z., Seng J., Cao Z., Gu Y., Han M. (2019). EZH2 Contributes To Cisplatin Resistance In Breast Cancer By Epigenetically Suppressing MiR-381 Expression. OncoTargets Ther..

[45] Yu Y., Qi J., Xiong J., Jiang L., Cui D., He J., Chen P., Li L., Wu C., Ma T. (2019). Epigenetic Co-Deregulation of EZH2/TET1 Is a Senescence-Countering, Actionable Vulnerability in Triple-Negative Breast Cancer. Theranostics.

[46] Zeng J., Zhang J., Sun Y., Wang J., Ren C., Banerjee S., Ouyang L., Wang Y. (2022). Targeting EZH2 for Cancer Therapy: From Current Progress to Novel Strategies. Eur. J. Med. Chem..

